# A Rapid LAMP-Based Method for Screening Poultry Samples for *Campylobacter* Without Enrichment

**DOI:** 10.3389/fmicb.2018.02401

**Published:** 2018-10-15

**Authors:** M. Rosario Romero, Nigel Cook

**Affiliations:** Fera Science, Ltd., York, United Kingdom

**Keywords:** food safety, poultry, carcasses, *Campylobacter*, detection, LAMP, internal amplification control

## Abstract

*Campylobacter* is the most prominent bacterium associated with foodborne disease and the majority of human infection cases are attributed to chicken. Rapid methods capable of determining the *Campylobacter* status of poultry products in a short time are needed in today’s fast-paced food supply chain. In this study, we developed and evaluated an easy to perform, rapid and robust method for direct detection of *Campylobacter* in poultry carcasses based on loop-mediated isothermal DNA AMPlification (LAMP). The method does not require bacterial culture or DNA purification and generates results in just an hour. A total of 171 swabs from chicken and turkey slaughter houses were analyzed in parallel by both LAMP and conventional culture-based enumeration methods to evaluate the performance of the rapid method. *Campylobacter* was detected by LAMP in 100% of swabs with an enumeration result of ≥800 cfu/swab, and 98.6% (69 out of 70) of samples reported as negative by enumeration (≤10 cfu/swab) were also negative by LAMP. The method is also suitable for analysis of boot swabs from poultry houses, and therefore it represents a convenient screening tool that can be implemented on farm, at slaughter houses, processing plants or retail, to help with the control of *Campylobacter* contamination throughout the food supply chain. The inclusion of an internal amplification control prevents any potential false negative results due to DNA amplification inhibitors that might be present in the sample.

## Introduction

*Campylobacter* is the most frequently reported cause of acute bacterial gastroenteritis in humans, with a large proportion of cases implicated with consumption of contaminated poultry products (Efsa Panel On Biological Hazards, 2015). A survey published by the United Kingdom Foods Standard Agency reported that over 60% of fresh chickens from major retailers in the United Kingdom analyzed between July 2015 and March 2016 were positive for *Campylobacter* and 11% showed contamination at high levels (>1,000 colony forming units × g^−1^)^1^. Although these figures represented an improvement with respect to the previous year, the report concluded that the proportion of chicken contaminated with high numbers of *Campylobacter* is still considerable. In order to achieve further reductions in contamination levels, efficient control measures need to be implemented throughout the poultry production chain. Accurate and rapid testing methods are necessary to support *Campylobacter* control strategies.

Current systems to monitor *Campylobacter* incidence on the broiler farm or processing plant involve sending samples to the laboratory for analysis, and then waiting days for the results. Detection of foodborne pathogens by conventional culture methods is a reliable approach, but it requires specialist laboratories and several days to generate results, and therefore there is no real-time information on pathogen presence or absence. Rapid and robust testing systems are needed for implementation on-site to inform rapid action. Other methods for detection and identification of *Campylobacter* have been published in recent years (Velusamy et al., 2010), including antibody-based detection (Wadl et al., 2009), PCR (Josefsen et al., 2004; Leblanc-Maridor et al., 2011; de Boer et al., 2015), DNA microarrays (Quinones et al., 2007; Donatin et al., 2013), and loop-mediated isothermal DNA amplification (LAMP) (Yamazaki et al., 2009; Dong et al., 2014; Sabike et al., 2016). Nevertheless, most procedures are still not fully adequate to be deployed on-site, as they involve specialized skills or facilities to perform certain steps such as DNA extraction or enrichment culture.

A rapid, easy to perform assay should ideally work on crude samples with minimal preparation and sample handling, e.g., no culture-based enrichment or DNA extraction and purification. LAMP-based methods for *Campylobacter* have been produced in recent years. Eiken Chemical, Co., Ltd. (Tokyo, Japan) developed the Loopamp^®^ *Campylobacter* detection kit. This method requires specialized laboratory equipment, as well as pre-enrichment and therefore it is not suitable as a rapid method for on-site application. The Loopamp^®^ *Campylobacter* detection kit has previously been reported as useful for the analysis of chicken meat contaminated with *C. jejuni* and *C. coli* (Yamazaki et al., 2009). The study showed that LAMP achieved 98.5% sensitivity compared with conventional culture tests, and results were obtained in 23.5 to 25.5 h from the start of the enrichment culture, in contrast with the 3–4 days required for the conventional method. However, that approach still involved an enrichment step and a laborious three-step centrifugation protocol. Another research group (Dong et al., 2014) developed a LAMP assay for detection of *C. jejuni* in cattle farm samples with a limit of detection of 400 fg genomic DNA per test tube (approximately 226 genome copies). This assay is rapid, but culture-based sample enrichment was still needed for application to farm samples.

We previously developed a LAMP assay for the detection of themotolerant *Campylobacter* spp. (Romero et al., 2016) in poultry boot swabs. The procedure involved the use of immunomagnetic beads to isolate the bacteria from boot swab samples followed by detection via LAMP. Since then, a new DNA polymerase has become available that is claimed by the manufacturer to possess improved robustness and provide faster amplification rates (GspSSD LF DNA Polymerase, Optigene, Ltd., United Kingdom). In the present work, we have modified our previous assay with this new enzyme to develop a rapid method for the direct detection of *Campylobacter* in poultry carcass swabs. The procedure is performed directly on carcass swab samples without culture enrichment, bacterial isolation, or DNA purification, and results can be generated within 1 h of sample collection.

## Materials and Methods

### Bacterial Strains and Cultures

*Campylobacter jejuni* NCTC 11168, and *C. coli* NCTC 11350 were used in this study. *Campylobacter* stocks were stored at −20°C and cultured at 41.5 ± 1°C on charcoal cefoperazone desoxycholate agar plates (Oxoid, Basingstoke, United Kingdom) for 48 h under microaerobic conditions (5% O_2_, 10% CO_2_, 85% N_2_).

### Carcass Swabs

Carcass sponge swab kits (TS/15-BPW, Technical Service Consultants) were used. They are provided pre-wetted in 10 mL of buffer. Poultry carcass swabs were obtained from Faccenda Foods, Ltd., United Kingdom and Bernard Matthews Foods, Ltd., United Kingdom. Duplicate samples were taken from each bird by holding two swabs side by side and swabbing over the whole carcass (chickens) or over the breast (turkey). One of the duplicate swabs was delivered to our laboratory and either stored at −20°C or tested immediately by LAMP, and the other one was tested by the supplier using culture methods. For artificial contamination experiments, the supplier tested for the presence/absence of *Campylobacter* using a culture-based method based on ISO/TS 10272-1:2006 (Anonymous, 2006a). Only swabs from birds testing *Campylobacter*-negative were used for subsequent artificial contamination experiments. For analysis of naturally contaminated carcasses, the supplier tested samples according to an enumeration protocol based on ISO/TS 10272-2:2006 (Anonymous, 2006b).

For comparison of results in samples treated by SonoSteam^®^^2^, carcasses from two different broiler flocks were analyzed, 15 carcasses from each before treatment, just after evisceration, and 15 after the SonoSteam^®^ and air chilling processes. Duplicate swabs from each carcass were analyzed, respectively, by the supplier using bacterial enumeration and by Fera using LAMP.

### Boot Swabs

Boot swabs from turkey houses were provided by Bernard Matthews, Ltd., United Kingdom. Samples were collected in duplicate by walking through turkey sheds wearing boot socks. The boot socks were then bagged individually and sent for analysis. One of the duplicate swabs was delivered to our laboratory and either stored at −20°C or tested immediately by LAMP. The second duplicate was tested by the supplier by *Campylobacter* culture methods as above, using 25 g of boot swab plus litter. Enumeration results were provided as cfu × g^−1^.

### Extraction of Bacteria From Carcass Swabs

For LAMP testing, the swab was palpitated repeatedly (approximately 10 times) to elute the attached material. A small aliquot of the fluid (around 500 μl) was treated at 85°C for 5 min in a dry hot block. Five μl was then taken and added directly into a LAMP reaction tube.

For the culture methods, each carcass swab was resuspended in 10 mL of buffer and 1 mL was used. Enumeration results were back-calculated to cfu per swab.

### Preparation of Boot Swabs for LAMP

Boot swabs were re-suspended in 100 mL of phosphate buffer saline (PBS) + 0.01% Tween 20 (PBST) and shaken for 30 s. An aliquot of the suspension was diluted fourfold in PBST and then 500 μl of this was mixed with an equal volume of 0.3 M KOH and heated at 85°C for 5 min in a dry hot block. Five μl was then added to the LAMP reaction tube.

### Detection of *Campylobacter* by LAMP Assay

**FIGURE 1:**
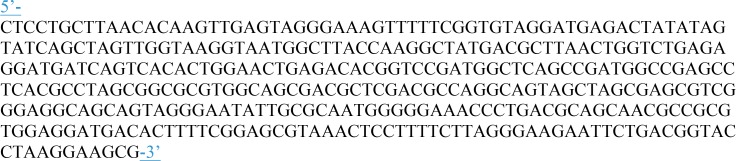
DNA sequence of the Internal Amplification Control.

The LAMP assay was described previously (Romero et al., 2016). It targets a region of the thermotolerant *Campylobacter* spp. 16S RNA gene, and was used with the following modifications: ISO-004 Isothermal Mastermix (OptiGene, Ltd., Horsham, United Kingdom) and a newly designed internal amplification control (IAC) were used in the reaction; this IAC was designed to have a more pronounced annealing temperature difference to the target than that used in the previous assay, and therefore to be more readily distinguishable from the target amplicon (**Figure 1** shows the IAC sequence). Oligo Calc: Oligonucleotide Properties Calculator^3^ was used to estimate the annealing temperature of several modifications of the region between the FIP and BIP primers. Four different versions of the IAC were designed, synthesized by Eurofins MWG Operon (Ebersberg, Germany) and tested. The IAC sequence producing the best discrimination from the target during annealing was selected and optimized as published previously (Cook et al., 2013). The optimal concentration for the LAMP assay was 340 IAC copies per reaction.

The master mix (15 μl), primers and IAC were combined in a 20 μl volume per reaction, and 5 μl of sample was added. **Table 1** details primer sequences and concentrations used. LAMP assays were performed in a Genie II (OptiGene, Ltd., Horsham, United Kingdom) using eight-tube strips. Every individual run included a positive control (purified *Campylobacter* DNA) and a negative control (water). The reactions were run at 65°C for 60 min. Amplicon annealing profiling was performed by heating to 98°C then cooling to 80°C at a rate of 0.05°C × sec^−1^.

**Table 1:** *Campylobacter* LAMP primers.

Primer name	Sequence (5′–3′)	Concentration in LAMP (nM)	Reference
OT1559	CTGCTTAACACAAGTTGAGTAGG	200	Uyttendaele et al., 1995
18-1rev	TTCCTTAGGTACCGTCAGAA	200	Lubeck et al., 2003
16SthCampyFIP	GGACCGTGTCTCAGTTCCAGTGTGACGGATGAGACTATATAGTATCAGCTAG	2000	Romero et al., 2016
16SthCampyBIP	CGGGAGGCAGCAGTAGGGAATATTGCTAAGAAAAGGAGTTTACGCTCCG	2000	Romero et al., 2016
16SthCampyF-Loop	GTTAAGCGTCATAGCCTTGGTAA	1000	Romero et al., 2016
16SthCampyB-Loop	GCGTGGAGGATGACACTT	1000	Romero et al., 2016


For boot swabs, ISO-004LNL Isothermal Mastermix (OptiGene, Ltd., Horsham, United Kingdom) was used, which neutralizes the alkaline solution used to assist bacterial lysis. The rest of the procedure was as described above.

### Purification of *Campylobacter* Genomic DNA

Genomic DNA was purified from 100 mL of *Campylobacter* culture using a DNA extraction kit (QIAGEN, Manchester, United Kingdom). A Qubit 4.0 fluorometer (Thermo Fisher Scientific, Wilmington, DE, United States) was used to measure the concentration and purity of the DNA. Purified DNA (1 μl/reaction) was used as a positive control in every LAMP experiment.

### Detection Limit of the Method Using Artificial Contamination

Broth cultures of *C. coli* were grown overnight. An aliquot of each culture was used for enumeration and the remainder was kept under hypoxic conditions at room temperature for 2 days, when the number of colony forming units (cfu) was determined. Serial dilutions were then made in maximum recovery diluent (MRD, Oxoid, Basingstoke, United Kingdom). One mL of each serial dilution, containing between 1.0 × 10^3^ and 2.3 × 10^5^ cfu, was added to *Campylobacter*-free turkey carcass swabs, which were then analyzed by the LAMP-based method. A total of 23 swabs were spiked in five independent experiments. Non-spiked swabs were also included as negative control.

## Results

### Performance of the IAC

**FIGURE 2:**
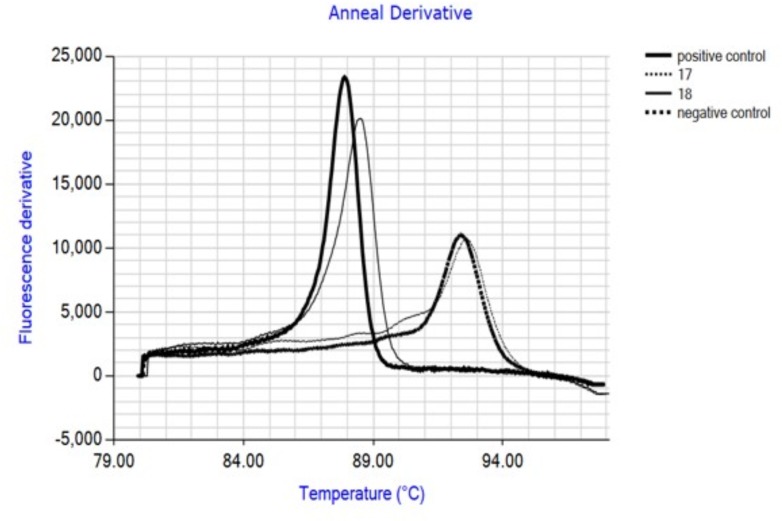
Annealing plots obtained after LAMP amplification showing discrimination of IAC and target. Internal amplification control (IAC) was included in all samples. Positive control: purified *C. coli DNA*; 17: carcass swab not contaminated with *Campylobacter*; 18: carcass swab contaminated with *Campylobacter*; negative control: water.

The IAC produces an annealing derivative with a peak at 92.5°C, clearly distinguishable from that of the target amplicon (88°C). Negative samples are easily detected by the presence of a single annealing peak at 92.5°C (**Figure 2**).

### Limit of Detection of the Method, Using Artificially Contaminated Swabs

The analysis of turkey carcass swabs spiked with different amounts of *C. coli* showed that the method could consistently detect down to 10^4^ cfu per swab, and in 2/5 tests down to 10^3^ cfu (**Table 2**).

**Table 2:** LAMP-based detection of *C. coli* on turkey carcass swabs at various artificial contamination levels.

Contamination level (cfu/swab)	Number of samples that tested *Campylobacter*-positive
10^5^	5/5^∗^
10^4^	5/5^∗^
10^3^	2/5^∗^
10^2^	0/4^∗^
10^1^	0/4^∗^
Uncontaminated	0/4^∗^

*^∗^Number of samples tested at the corresponding contamination level.*

### Detection of *Campylobacter* in Naturally Contaminated Chicken and Turkey Carcasses

**Table 3** shows the culture enumeration results provided by the supplier’s testing laboratories alongside the results obtained with the LAMP-based method. The LAMP based method could consistently detect contamination at levels of 800 cfu per swab and higher; however, the probability of detection diminished as the counts per swab decreased, resulting in some false negative results in swabs containing 10-760 cfu. Out of 70 samples that tested negative in the enumeration assay, only one generated a positive result by LAMP.

**Table 3:** Detection of thermophilic *Campylobacter* spp. in naturally contaminated chicken and turkey carcasses, by a culture-based enumeration method and by the LAMP-based method.

		LAMP results		
Enumeration results (CFU/swab)	Number of swabs	Positive swabs	Negative swabs	Percentage positive samples by LAMP
≥800	36	36	0	100
400–760	11	5	6	45.4
200–380	14	4	10	28.6
100–190	22	3	19	13.6
10–80	18	0	18	0
0^∗^	70	1	69	1.4
Number of swabs	171	50	121	

*^∗^Absence of colonies on the culture plates was reported as <10 cfu/swab, as the limit of detection of the method is 1 cfu × mL^−1^ and samples were diluted 10-fold before plating.*

One of the slaughter houses that supplied chicken carcass swabs for this study uses a combination of steam and ultrasound (SonoSteam^®^) to reduce *Campylobacter* loads on the carcasses. To explore the effectiveness of the LAMP approach in such a setting, samples were collected before and after SonoSteam^®^ treatment from two separate broiler flocks (one positive and one negative for *Campylobacter*, based on farm results). Duplicate swabs from each carcass were analyzed by both enumeration of *Campylobacter* spp. and LAMP, respectively. Results are shown in **Table 4**. All pretreatment samples from the *Campylobacter*-positive flock tested positive by the LAMP-based method, while 14/15 post-treatment samples (ranging from 0 to 410 cfu/swab) were negative for *Campylobacter* by LAMP. All samples from the *Campylobacter*-negative flock tested negative by LAMP, although the enumeration method detected low numbers of cfu/swab in the pre-treatment set.

**Table 4:** Detection of thermophilic *Campylobacter* spp. in broiler carcasses before and after SonoSteam^®^ treatment by culture-based enumeration and by the LAMP-based method.

*Campylobacter*-positive flock					
**Pre-SonoSteam**			**Post-SonoSteam/chill**		
**Sample number**	**LAMP result**	**Enumeration result (cfu/swab)**	**Sample number**	**LAMP result**	**Enumeration result (cfu/swab)**
1	Positive	1300	16	Negative	30
2	Positive	3600	17	Negative	130
3	Positive	5300	18	Negative	340
4	Positive	1800	19	Negative	90
5	Positive	3100	20	Negative	<10
6	Positive	2600	21	Negative	350
7	Positive	5200	22	Negative	600
8	Positive	3600	23	Positive	120
9	Positive	5100	24	Negative	180
10	Positive	2100	25	Negative	40
11	Positive	2400	26	Negative	90
12	Positive	4700	27	Negative	140
13	Positive	7800	28	Negative	30
14	Positive	1600	29	Negative	480
15	Positive	2100	30	Positive	410
***Campylobacter*-negative flock**		
31	Negative	30	46	Negative	<10
32	Negative	120	47	Negative	<10
33	Negative	220	48	Negative	<10
34	Negative	170	49	Negative	<10
35	Negative	40	50	Negative	<10
36	Negative	250	51	Negative	<10
37	Negative	70	52	Negative	<10
38	Negative	360	53	Negative	<10
39	Negative	80	54	Negative	<10
40	Negative	10	55	Negative	<10
41	Negative	190	56	Negative	<10
42	Negative	100	57	Negative	<10
43	Negative	640	58	Negative	<10
44	Negative	480	59	Negative	<10
45	Negative	60	60	Negative	<10

### Detection of *Campylobacter* in Naturally Contaminated Turkey Boot Swabs

**Table 5** shows the information obtained when a small set of duplicate turkey boot swabs were tested by the LAMP-based method and by a culture-based method, respectively. LAMP could identify *Campylobacter* contamination at levels of 30 cfu × g^−1^ and above.

**Table 5:** Detection of thermophilic *Campylobacter* spp. in naturally contaminated turkey boot swabs, tested by a culture-based enumeration method and by the LAMP-based method, respectively.

Swab number	Culture enumeration results (cfu/g)	LAMP results
1	<10	Negative
2	<10	Negative
3	<10	Negative
4	1	Negative
5	10	Negative
6	20	Negative
7	30	Positive
8	100	Positive
9	72,000	Positive
10	Spread^∗^	Positive

*^∗^Spread: plates were overgrown and individual colonies could not be counted.*

## Discussion

Due to the impact of *Campylobacter* on human health, governments and industry have been developing strategies to reduce the levels of *Campylobacter* contamination in poultry, the main vehicle of *Campylobacter* food poisoning for humans. Testing poultry products for the presence of *Campylobacter* is key to monitor the effect of biosecurity measures and to reduce the risk of contaminated products reaching the consumer. In this study, a rapid, robust, and easy to perform method has been optimized and evaluated against standard enumeration methods. The method uses LAMP technology to detect *Campylobacter* spp. directly in crude suspensions of carcass swabs, without pre-enrichment or DNA purification.

The *Campylobacter* LAMP assay has a probability of detection of 95% with 22 genome copies when performed with pure DNA using *Tin* Isothermal Mastermix (OptiGene, Ltd., Horsham, United Kingdom) (Romero et al., 2016). In the method described here, a sponge swab pre-wetted with 10 mL of buffer is used, and after homogenization, the fluid is squeezed out of the sponge and used as template for the reaction. When swabs were artificially contaminated with defined numbers of *Campylobacter* colony forming units, the limit of detection of the method was between 10^3^ and 10^4^ cfu per swab. Since the total volume of fluid per swab is 10 mL and the volume tested in a single reaction is 5 μl, the *Campylobacter* levels detected are equivalent to 0.5 and 5 cfu per reaction, respectively. This is lower than the detection levels observed with pure DNA previously suggesting that dead cells were present in the cultures used to spike the swabs. These would not be detected by a culture method, so they would not have been accounted for during enumeration, but would still give a positive signal by LAMP if present at sufficient levels.

The analysis of naturally contaminated samples showed that the LAMP-based method detected *Campylobacter* in all swabs from carcasses with enumeration results of 800 cfu/swab or greater. For samples with enumeration results below 800 cfu/swab, the overall probability of detection of the LAMP assay decreases gradually as the counts per swab decrease, resulting in some false negative results. This is expected, since the LAMP test is performed on a 5 μl aliquot of a 10 mL swab suspension, which on average equates to less than one cfu per reaction at 800 cfu/swab. Despite this, some of the samples with colony counts below 800 tested positive by LAMP. This will be explained by the presence of dead or non-culturable *Campylobacter*, which will only be detected by LAMP.

Out of 70 carcasses for which no colonies were obtained by the culture method, only one was detected as positive by LAMP. In addition, a further set of 60 turkey duplicate swabs were tested in parallel by the LAMP method and by the enrichment culture method that establishes presence/absence of *Campylobacter* (Anonymous, 2006a) (data not shown). All swabs gave a negative result by both methods, confirming that the likelihood of obtaining false positives by the LAMP-based method is very low.

Whilst with artificially contaminated carcass swabs the method consistently produced positive LAMP results with 10^4^ cfu/swab, in naturally contaminated samples all swabs with ≥800 cfu/swab tested positive by LAMP. Since the culture-based method detects only viable cells whereas LAMP detects also dead cells, this apparent discrepancy suggests that carcasses that are contaminated with high levels of *Campylobacter* normally contain high numbers of dead bacteria, more so than those that might occur in the pure bacterial cultures used for spiking experiments.

A fundamental difference between DNA amplification methods and culture-based methods is that the latter naturally have higher sensitivity, since the bacteria are incubated for a period of time and allowed to multiply until visible colonies are obtained. However, rapid results at the expense of some sensitivity may be acceptable for certain applications, such as routine screening. The method described here has a limit of detection for poultry carcass of 800 cfu/swab under the conditions applied and with the specific sampling and sample preparation protocols used in this study. However, modifications can be made to these procedures to achieve adequate detection levels, since the LAMP reaction itself is highly sensitive. Should higher sensitivity be required, various adjustments could be explored, such as reducing the volume of buffer the sample is collected in or concentrating the sample, e.g., by centrifugation and resuspension in a smaller volume.

Our original method (Romero et al., 2016) was developed for the analysis of poultry boot swabs, and involved immunoseparation of the bacteria from swab suspensions followed by LAMP using the Tin enzyme (ISO-001 Tin, OptiGene, Ltd., Horsham, United Kingdom). A small set of turkey boot swabs were tested in the current investigation to assess the performance of the new direct LAMP procedure in these samples, which carry fecal and other materials (bedding, feathers) and might pose a bigger analytical challenge. Although the number of samples is small, these preliminary results show a similar trend to those from carcass swabs, suggesting that the method is robust enough to be applicable to different types of samples, including very complex materials that would inhibit other molecular reactions such as PCR.

A recent publication (Sabike et al., 2016) reported the use of *Campylobacter* LAMP assay for direct detection in chicken fecal samples. They achieved detection of 3.89 and 3.60 log cfu × g^−1^ of fecal sample for *C. jejuni* and *C. coli*, respectively, in artificially contaminated fecal samples, and a three-step DNA precipitation protocol was used to improve sensitivity. In contrast, the approach presented here does not require DNA precipitation, making it faster and simpler to perform. The only equipment needed is a hot block and a small and portable battery powered instrument to perform the reaction and read-out of results. Thus, our method is suitable for rapid screening of *Campylobacter* in different settings including processing plants and poultry farms. The method has also been successfully tested in cattle carcass swabs, with and without artificial contamination (data not shown), suggesting that as expected, it can be applied in settings other than poultry production.

Sabike et al. (2016) reported that their analysis of naturally contaminated specimens from one particular farm showed a high frequency of false negative results for *Campylobacter* loadings ranging from <3 to 5.81 log cfu × g^−1^. The authors discussed that this high rate of false negatives may be explained by inhibitors of DNA amplification, possibly derived from substances in the poultry feeding stuffs. Indeed, in our preliminary investigation with boot swabs (fecal material) a difference in method performance between different poultry farms was also observed. To deal with potential inhibitors frequently present in complex matrices such as food and fecal samples, our assay includes a competitive IAC (Hoorfar et al., 2004). The presence of inhibitors of DNA amplification in a sample may result in false negative results. However, in our assay, if DNA amplification has taken place, a signal will always be produced, and the temperature of the annealing peak will indicate if it is a *Campylobacter*-positive or a true negative result. The IAC is also a useful tool for assay optimization; in the absence of target amplification, the IAC fails to amplify it is an indication that the reaction has not proceeded and changes to the sample preparation can be tested. This was applied in our trials with boot swabs and it helped to establish that a fourfold dilution of the swab suspension produced optimal results.

The direct LAMP-based method was applied to carcasses that had been treated with a combination of steam with ultrasound (SonoSteam^®^) at slaughter to reduce *Campylobacter* loads. As observed previously (Musavian et al., 2014), treatment with SonoSteam^®^ significantly reduced viable *Campylobacter* levels on chicken carcasses (average reduction of 1.4 log_10_ cfu in the sample set used in this study). The results obtained with LAMP are similar to those obtained with the culture-based method in that in most post-treatment samples a reduction of *Campylobacter* load is indicated. Therefore, the direct LAMP method is suitable for monitoring *Campylobacter* levels in plants where poultry carcasses have been treated by SonoSteam^®^ or similar decontamination procedures, and to ensure that high level contamination is eliminated. Nevertheless, the results obtained with samples post-treatment indicate that low residual levels of *Campylobacter* that would be detected by culture methods may result in false negatives by LAMP due to the limit of sensitivity of the method. As an additional observation, the absence of a LAMP signal in most post-treatment samples suggests that the SonoSteam^®^/air chilling procedure is not simply killing *Campylobacter*, in which case dead cells on the carcass surface would be detected by LAMP and more positive results would be expected. Instead, the treatment might be removing the bacteria from the carcass surface or damaging their DNA so that it can no longer be amplified by LAMP.

The United Kingdom government/industry set a target to reduce the prevalence of chickens containing greater than 1000 cfu per g skin, considered the most contaminated, to below 10% at the end of the slaughter process^4^; it was considered that achieving this target should consequently reduce the prevalence of *Campylobacter* in the food chain. A survey to determine progress toward this target was carried out in 2016, analyzing chickens sold at retail using a culture-based method^5^. The LAMP-based method presented here is suitable for deployment not just at point of sale, but also before and immediately after the slaughter process, and thus closer to the stages where the *Campylobacter* colonization status of a bird is determined. The colonization status of a chicken is recognized as the most important factor affecting numbers of *Campylobacter* cfu on a carcass (European Food Safety Authority [EFSA], 2010), and therefore the ability to assess this as soon as conveniently possible will be highly useful to allow meat inspectors/slaughter house/processing plant personnel rapid logistic decisions. It could also help to monitor the effectiveness of biosecurity measures implemented to control *Campylobacter* contamination. The LAMP-based system described furthermore offers scope for automation and indeed preliminary work is being carried out in this respect (unpublished data).

This study reports validation data on the performance of a novel rapid method for *Campylobacter* detection on poultry carcasses as compared with conventional ISO-based culture methods. Further validation work will be conducted to provide more information about the equivalence of the method to ISO standards for different purposes and to establish how the rapid method could be integrated into surveillance and enforcement activities and thus contribute to food safety. The method in its present state of development has the acknowledged limitation of a lack of sensitivity below 800 cfu per swab, which will result in some analyses producing negative results if the contamination level is below that figure. However, it must be stressed that the sampling protocol used in this study for both LAMP and culture (half or whole carcass swabbing) is different to that used in the ISO culture method on which current United Kingdom and EU recommendations are based (cutting out a small piece of skin). Some of the carcasses from this study that resulted in less than 800 cfu per swab (whole chicken carcass swabbed) may have also produced a negative result by culture following the standard method that analyses only 10–25 g of skin. Therefore, the sensitivity of the method is sufficient for detection of the *Campylobacter* contamination level (1000 cfu × g^−1^ skin) which is a process hygiene criterion recommended in European regulation^6^. It has been estimated (Efsa Panel On Biological Hazards, 2011) that full compliance with this criterion by the poultry production industry would lead to a public health risk reduction of over 50%. The LAMP-based method can therefore be a useful tool for rapid screening of poultry carcasses at primary production, assisting in the management of foodborne *Campylobacter* transmission.

## Author Contributions

MR contributed design and coordination of the study, testing by LAMP and data analysis and interpretation. NC contributed to the LAMP assay design and data interpretation. Both authors contributed to manuscript writing.

## Conflict of Interest Statement

The authors declare that the research was conducted in the absence of any commercial or financial relationships that could be construed as a potential conflict of interest.

## Footnotes

1 Foods Standards Agency, United Kingdom, 2018. https://www.food.gov.uk/research/foodborne-diseases/a-microbiological-survey-of-campylobacter-contamination-in-fresh-whole-uk-produced-chilled-chickens-at-retail-sale-y234.

2 https://sonosteam.com/

3 http://biotools.nubic.northwestern.edu/OligoCalc.html

4 Foods Standards Agency, United Kingdom, 2013. https://acmsf.food.gov.uk/sites/default/files/multimedia/pdfs/board/board-papers-2013/fsa-130904.pdf.

5 Foods Standards Agency, United Kingdom, 2018. https://www.food.gov.uk/research/foodborne-diseases/a-microbiological-survey-of-campylobacter-contamination-in-fresh-whole-uk-produced-chilled-chickens-at-retail-sale-y234.

6 https://publications.europa.eu/en/publication-detail/-/publication/218f322f-8890-11e7-b5c6-01aa75ed71a1
